# Reliability of Phase Velocity Measurements of Flexural Acoustic Waves in the Human Tibia *In-Vivo*

**DOI:** 10.1371/journal.pone.0152417

**Published:** 2016-03-25

**Authors:** Florian Vogl, Karin Schnüriger, Hans Gerber, William R. Taylor

**Affiliations:** Institute for Biomechanics, Department of Health Sciences and Technology, Swiss Federal Institute for Technology in Zurich, Zurich, Switzerland; Monash University, MALAYSIA

## Abstract

**Purpose:**

Axial-transmission acoustics have shown to be a promising technique to measure individual bone properties and detect bone pathologies. With the ultimate goal being the *in-vivo* application of such systems, quantification of the key aspects governing the reliability is crucial to bring this method towards clinical use.

**Materials and Methods:**

This work presents a systematic reliability study quantifying the sources of variability and their magnitudes of *in-vivo* measurements using axial-transmission acoustics. 42 healthy subjects were measured by an experienced operator twice per week, over a four-month period, resulting in over 150000 wave measurements. In a complementary study to assess the influence of different operators performing the measurements, 10 novice operators were trained, and each measured 5 subjects on a single occasion, using the same measurement protocol as in the first part of the study.

**Results:**

The estimated standard error for the measurement protocol used to collect the study data was ∼ 17 m/s (∼ 4% of the grand mean) and the index of dependability, as a measure of reliability, was Φ = 0.81. It was shown that the method is suitable for multi-operator use and that the reliability can be improved efficiently by additional measurements with device repositioning, while additional measurements without repositioning cannot improve the reliability substantially. Phase velocity values were found to be significantly higher in males than in females (*p* < 10^−5^) and an intra-class correlation coefficient of *r* = 0.70 was found between the legs of each subject.

**Conclusions:**

The high reliability of this non-invasive approach and its intrinsic sensitivity to mechanical properties opens perspectives for the rapid and inexpensive clinical assessment of bone pathologies, as well as for monitoring programmes without any radiation exposure for the patient.

## Introduction

The clinical assessment of bone pathologies (e.g. osteoporosis) and overall fracture risk is a complex challenge, requiring the evaluation of a multitude of material and structural bone properties. While current standard radiation based methods to assess bone are sensitive to only a very limited set of such properties, acoustic methods are intrinsically suitable for probing mechanical properties over different length scales. Dual-energy x-ray absorptiometry (DEXA), which is the current gold-standard in the clinical diagnosis of osteoporosis, only provides information about the mineralization and projected geometry of the bone. Even 3D-methods such as quantitative-computed tomography (qCT) are still limited to the characterization of only the inorganic component of the bone.

Axial transmission quantitative ultrasound (ax-QUS) is a bone sonometry technique to assess the properties of cortical bone in the human body in rapid, non-invasive, and radiation- free fashion. Primary application sites of this technique include the long bones of the body, particularly the tibia, ulna, and the radius [1]. In ax-QUS, transducers are placed along the bone to be examined and transmit an ultrasonic wave into the cortical layer through the overlying soft-tissues. Separate surface sensors measure the propagation of this wave, which provides key information on bone properties such as cortical thickness, elastic modulus, porosity, or bending stiffness. Advantages of this technique include the requirement for only unilateral access to the bone and its ability to characterize a multitude of properties over a large region of the bone, even within a single measurement.

Early ax-QUS devices analysed the “first arriving signal” (FAS), sometimes also termed the “speed of sound”, which was thought to be sensitive to the properties of the medium. Commercial ax-QUS devices working at around 250 kHz and 1 MHz have been developed based on this idea and have demonstrated a certain level of sensitivity to changes in cortical properties associated with renal disease [2] or Crohn’s disease [3]. However, the sensitivity of FAS approaches to DEXA-defined osteoporosis was shown to be low [4, 5].

It was later found that the FAS consisted of not only one but rather multiple waves with different properties propagating within the bone at the same time. These different waves are now known to contribute to the measured FAS depending on the geometry of the bone, the cortical thickness d, and the wavelength λ of the wave, making valid interpretation of FAS results difficult [6]. Additional numerical and experimental studies have found that the waves inside long bones can be well described by plate- and tube-like theories [7, 8, 9], where the FAS was identified to correspond to a lateral head wave for λ << d and to a S0-like guided wave for λ > d. Also the energetic-late-arrival (ELA), a high energy signal arriving after the FAS observed in earlier studies, was identified to correspond to a Rayleigh wave for λ << d and to a A0-like guided wave for λ > d.

After the identification of the frequency’s importance, considerable effort has been put into assessing the potential information accessible through different frequency ranges in *in- vitro* [10, 9] as well as *in-vivo* studies [11, 12, 13]. These works demonstrated that the comprehensive analysis of guided waves with specific frequencies, which goes beyond the simple analysis of the FAS, osteoporosis [11, 12, 13], cortical thickness [14, 9] and porosity [1, 15]. By examining the frequency range from 250 kHz to 1.25 MHz, these experiments clearly showed that low-frequency waves are necessary to access geometrical properties such as the cortical thickness, as well as properties at or near the endosteum. On the contrary, such properties are inaccessible using high-frequency waves due to their limited penetration depth into the bone. Despite the clear advantages of using low-frequency waves, most experiments were restricted in measurement frequency due to technical limitations, and the possibilities of low-frequency regimes below 250 kHz remain largely unexplored, with the few available studies indicating the possibility to measure bone mass density [16] and training effects [17]. At these low frequencies, where the wavelength is in the cm range, the wave is expected to be highly dependent on the bone’s geometry, cortical thickness, and homogenized bone properties across the whole cross-section. Furthermore, since the attenuation of bone increases with frequency [18], usage of low frequency waves should allow for an improved signal-to-noise ratio.

The *in-vivo* application of wave transmission techniques remains challenging as a multitude of factors including soft-tissue artefact, operator performance, and probe placement can all directly influence the quality and repeatability of the measurements. While a bidirectional technique has been developed to reduce soft-tissue effects [1], no conclusive quantitative in- formation on the influence of the other factors exists to date. A study that aimed to address this question remained inconclusive due to the insufficient amount of data collected (10 subjects, 2 occasions, 2 operators), showing the need for a systematic, high data approach to estimate the sources and magnitudes of variability satisfactorily [19]. In order to optimise applicability and efficiency for clinical applications, a quantification of the dominant factors affecting reliability is clearly required.

With the vision of taking advantage of the beneficial characteristics of low-frequency waves for the non-invasive assessment of bone properties and diagnosis of bone pathologies, this study aimed to quantify the precision and the sources of variability of the axial wave transmission measurements, including the inter-subject and inter-leg variability, as well as variability caused by probe repositioning, operator performance, and multiple consecutive measurements.

## Methods

### Study design

In order to assess the precision of axial transmission wave measurements using the Bone Stiffness Measurement Device (BSMD), as well as estimate the contributions of different factors to the variability, this study consisted of a) an intra-operator study and b) an inter-operator study.

**Fig 1:**
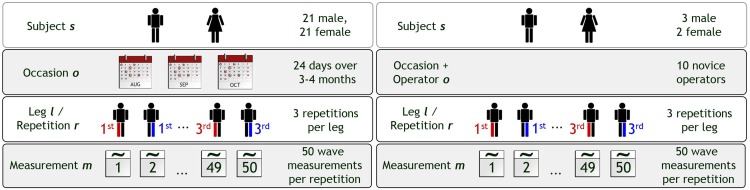
Study Design. Schematic representation of the study design of the intra-operator (left) and inter-operator study (right).

In the intra-operator study, one experienced operator measured 42 healthy subjects (21 males, aged 27.0 ± 3.2 years; 21 females, aged 28.6 ± 5.6 years) twice per week, over a period of three to four months, resulting in 24 measurement occasions per subject (Fig 1).

In the inter-operator study, 10 inexperienced operators were trained in the use of the device, before measuring the same 5 healthy subjects (3 male/2 female, aged 28.2 ± 1.6 years) on a single measurement occasion (Fig 1). The training included studying a 15-page manual and a collective 2-hour demonstration session, presented by an experienced operator. The order of subject measurements was randomized for each single operator.

All subjects received extensive verbal and written information, and also provided written consent before enrolling in this study. The ethical approval for this project was granted by the Ethics Commission of the ETH Zurich (reference number: EK 2013-N-75).

Excluded from the study were persons who had suffered a knee or tibia fracture within the previous year, persons with prostheses or implants in the knee, leg, or foot, persons suffering from bone diseases, and persons under medical treatment or medication (except contraceptives). The operators all had technical or scientific backgrounds in fields not related to this project or scientific field. During the intra-operator study one female participant withdrew from the study due to slight bruising from the sensor fixation pressure. The measurements from this subject were therefore not included in the statistical analysis. During the inter-operator study, one male subject was withdrawn from the study due to an unrelated tibial fracture, so that two operators could not measure this subject.

### Bone Stiffness Measurement Device (BSMD)

**Fig 2:**
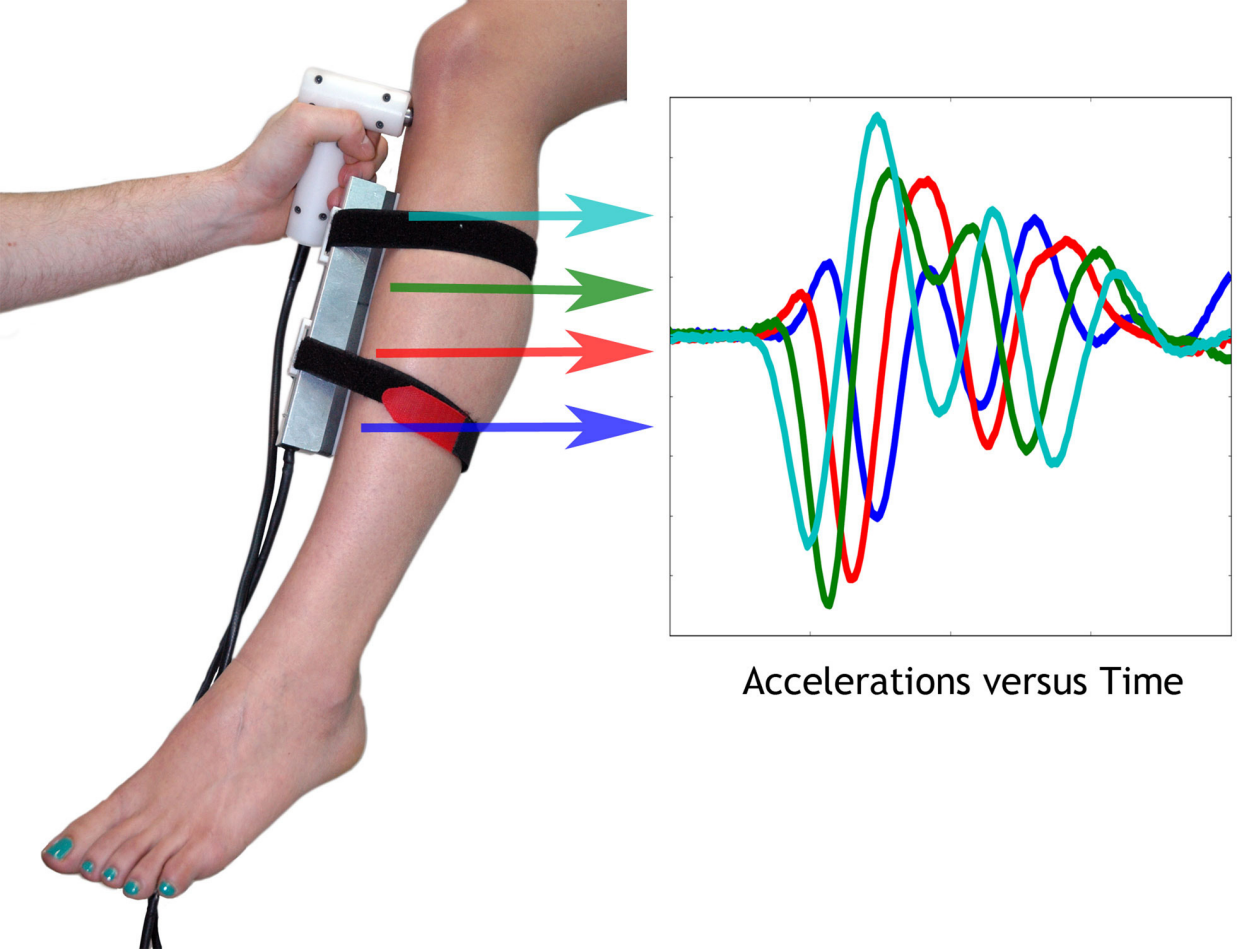
Bone Stiffness Measurement Device (BSMD). Left: A photograph of the BSMD measuring a subject and a schematic representation of the accelerations measured by the four sensors along the tibia.

The Bone Stiffness Measurement Device (BSMD) is a device to measure the velocity of waves propagating in long bones of the human body. It consists of three main parts: a piezo-electric actuator to create the wave, a custom made sensor with four accelerometers to measure the wave propagation, and a portable electronic unit to handle data acquisition and device control. Fig 2 shows the basic working principle and the application of the BSMD at the tibia.

The actuator consists of a P-840.20 piezo-stack (PI Ceramic GmbH, Lederhose, Germany) with a stainless steel half-sphere attachment-head, both mounted in a custom made teflon housing. A Gaussian enveloped sine wave with central frequency of 3 kHz and a Full-Width-Half-Maximum of 1 kHz drives the actuator after amplification through an E-617 high-power piezo-amplifier (PI GmbH & Co. KG, Karlsruhe, Germany). The wave propagation is measured using four 4516 accelerometers (Brüel & Kjael GmbH, Pöcking, Germany), mounted in a custom aluminium housing. The accelerometers are linearly arranged with an inter-sensor distance of 4 cm and are spring mounted to improve coupling with the bone in the presence of soft tissue.

The signal generation for the actuator and the data acquisition from the accelerometers are handled by a USB-4431 data acquisition card (National Instruments) with a sampling rate of 96 kHz and an acquisition length of 200 samples, which corresponds to more than 6 times the cycle length for the central frequency of 3 kHz. The device was controlled by a laptop running in-house software, programmed in Labview 13 (National Instruments, Austin, Texas), which handled the signal generation and storage of raw measurement files for off-line analysis.

To acquire the phase velocity from the four measured accelerations, the signals were windowed using a Gaussian window with a width of 150 μs, which removed the noise and reflections in the latter part of the signal while retaining the first full oscillations. All signals were normalized to their highest amplitude and were transformed into frequency space using a Fast-Fourier-Transform. The resulting phase information was unwrapped around the centre-frequency and ordered by taking into consideration the spatial arrangement of the sensors. The phase velocity v at frequency f = 3 kHz was calculated as follows:

$$v\left(f\right)=\frac{\Delta d\;2\pi \;f}{\Delta \phi },$$ (1)

where Δd is the distance and Δφ is the phase difference between two sensors respectively. The phase velocities between pairs of sensors had to be consistent for the measurement to be considered valid. The final value for the velocity was calculated from the average of the three adjacent sensor pairs.This phase-velocity based analysis approach was validated on 24 simple phantoms (tubes and beams of different materials) by comparison with results from standard Timoshenko for beams and Hermann-Mirsky theory for cylindrical shells.

### Measurement protocol

At the beginning of each measurement occasion, the operator palpated the malleolus medialis and the joint gap above the medial tibial condyle and marked the mid-point between these two landmarks along the facies medialis. This mark was then used to position the centre of the BSMD-sensor for all measurements taken during each measurement occasion.

The BSMD-sensor was fixed to the marked position on the left leg using two straps and the BSMD-actuator was applied at the tibial head and driven by a pulse (centre-frequency: 3 kHz, bandwidth: 1 kHz), creating a flexural wave inside the bone. The accelerations of the resulting wave were measured by the BSMD-sensor, digitized, and transferred to the laptop for off-line analysis. Fifty such wave measurements were taken in succession before transfer- ring the BSMD-actuator and BSMD-sensor to the other leg. Using this procedure, both legs were measured alternately three times each, yielding a total of 150 wave measurements per tibia per measurement occasion for each subject.

### Statistical Analysis

To assess the precision of the presented methodology and to quantify the main sources of measurement uncertainty, we performed an analysis of variance using Generalizability-theory (G-theory)[20]. This mathematical framework allows an estimation of the various sources and their contributions to variability using a reliability study (G-study in the terminology of the framework). With these variability contributions it was then possible to estimate the reliability of further study designs (D-studies) with the goal of finding and optimizing the measurement protocol. The assumed study design for both the intra- and inter-operator studies corresponded to a fully crossed s × l × o × r × m study design, where s denotes the subject, l the leg, o the occasion (confounded with the operator in case of the inter-operator study), r the repetition, and m the measurement (Fig 1).

Following the “analogous ANOVA procedure” for unbalanced designs, the different variance components were estimated using the software “urGENOVA” (version 2.1, University of Iowa) [20]. Using these estimated variance components, we estimated the reliability of different measurement procedures based on different numbers of occasions, repetitions, and measurements. Here, the number of occasions and repetitions were varied from one to five, while the number of measurements per repetition was kept at m = 50, and the operator was kept constant. Considering one leg of the subject as the object of measurement, the index of dependability Φ [20, 21] was calculated using:

$$\Phi =\frac{\mathrm{universe variance}}{\mathrm{total variance}}=\frac{\sigma_{s}^{2}+\sigma_{l}^{2}+\sigma_{\mathrm{sl}}^{2}}{\sigma_{\mathrm{total}}^{2}},$$ (2)

which is a measure for the reliability of absolute values. In the calculation of $\sigma_{total}^{2}$ as the sum of all variance components, negative estimates of variance components were set to zero. This procedure gives a biased but conservative estimate for the index of dependability. In order to access the measurement precision, the standard error SE of each individual D-study was calculated using

$$\mathrm{SE}=\sqrt{\mathrm{total variance}-\mathrm{universe variance}}=\sqrt{\sigma_{\mathrm{total}}^{2}-\sigma_{s}^{2}-\sigma_{\mathrm{sl}}^{2}},$$ (3)

which is the standard-deviation caused by all effects except the object of measurement, in this case the subjects and their inter-leg variability.

To assess the agreement between the mean phase velocity of both legs for each subject the one-way Intra-class correlation coefficient was calculated as

$$\mathrm{ICC}=\frac{\mathrm{BMW}-\mathrm{WMS}}{\mathrm{BMS}+\mathrm{WMS}},$$ (4)

where BMS is the between target mean square and WMS is the within target mean square [22].

## Results

The estimated variance components for the different effects were determined (Table 1), resulting in an estimated SE for each single wave measurement of 37 m/s which is 8% of the grand mean.

**Table 1:** Variance estimates.

Intra-operator study		Inter-operator study	
Effect	*σ*^2^	Effect	*σ*^2^
s	1015.09	s	1062.98
l	16.22	l	18.24
o	39.60	o	18.50
r	4.28	r	-6.49
m	0.12	m	1.07
sl	190.29	sl	78.27
so	103.84	so	95.72
sr	20.34	sr	15.43
sm	1.20	sm	2.11
lo	18.28	lo	31.90
lr	-2.82	lr	1.64
lm	0.27	lm	0.22
or	-21.78	or	32.34
om	1.43	om	6.46
rm	0.36	rm	1.84
slo	231.93	slo	291.99
slr	-2.42	slr	-33.88
slm	-3.48	slm	-4.11
sor	58.50	sor	-52.49
som	-10.93	som	-20.04
srm	-2.97	srm	-7.86
lor	15.50	lor	-39.40
lom	-3.55	lom	-7.81
lrm	-0.92	lrm	-1.34
orm	-6.09	orm	-13.24
slor	474.20	slor	554.07
slom	27.31	slom	30.75
slrm	14.58	slrm	13.37
sorm	51.16	sorm	55.89
lorm	13.71	lorm	18.14
**Grand mean**	475 m/s	**Grand mean**	480 m/s

Estimated variances σ2 for various effects i and the estimated grand mean for the intra-operator study (left) and the inter-operator study (right), where s is the subject, l is the leg, o is the occasion (intra-operator study) or the occasion/operator (inter-operator study), r is the repetition, and m is the measurement number.

A Mann-Whitney U test between the mean phase velocities of male and female subjects gave a p-value of p < 10^−5^. The ICC for the average phase velocity for both legs for each subject was ICC = 0.70. A Mann-Whitney U test between the pooled right vs the pooled left leg phase velocities gave a p-value of p = 0.36. After averaging the phase velocities for each occasion, the mean slope of the linear regressions of the phase velocities for each subject and each leg was *β*_*mean*_ = 0.16 m/s/day ± 0.33 m/s/day, indicating that there was no relevant drift over the course of the measurements.

**Fig 3:**
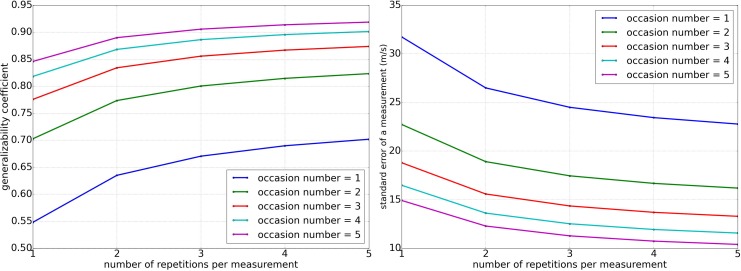
Index of reliability and standard error The estimated index of dependability (left) and standard error (right) calculated for a measurement resulting for various D-studies with different combinations of measurement occasions and measurement repetitions. The number of measurements without repositioning is m = 50.

The index of dependability varied between 0.55 and 0.92 for the different D-studies investigated, while the estimated SE varied between 32 m/s and 11 m/s (Fig 3).

## Discussion

Current clinical assessment of bone health is generally limited to radiation based approaches such as x-ray, DEXA, and CT, which are only able to provide metrics regarding the inorganic compounds of the bone, but are unable to directly access the mechanical properties of the bone. The dangers of radiation, together with the fact that not all hospitals possess the necessary equipment due to size and cost factors, makes regular and widespread screening using these methods problematic. Acoustic techniques offer a promising non-invasive approach for assessing bone that is intrinsically sensitive to the structure and mechanical properties of the bone, and which are able to complement current methods. Paired with the fact that the equipment is cheap and portable, these techniques are also a prime candidate for widespread screening applications. However, the *in-vivo* application of such acoustic techniques remains challenging due to a number of noise factors, such as soft tissue artefacts, device positioning, and operator performance, which are all thought to influence the quality of measurement. In this study, key factors that influence the reliability of acoustic wave measurements *in-vivo* have been addressed. Through quantification of the influence of subject, limb, operator, and repetition (with and without sensor repositioning), as well as combinations thereof, we have been able to demonstrate the key factors that govern the reliable use of axial transmission acoustics *in-vivo*, which is a critical requirement to be able to improve the approach and measurement protocols before successful translation into clinical settings.

The high value of $\sigma_{s}^{2}+\sigma_{l}^{2}+\sigma_{sl}^{2}$ shows that the largest variance in the measured phase velocity is attributable to the object of measurement itself, in this case the natural variation between different subjects and their legs. In comparison, the relatively low value of $\sigma_{l}^{2}$ and $\sigma_{s}^{2}$, together with the high ICC between legs, indicates that the wave velocity is dominantly governed by the subject, with only small differences between the two legs, arising from differences in the actual mechanical properties but possibly also from slightly different shapes and the resulting change in coupling to the sensors.

Comparison of the variance components $\sigma_{o}^{2}$ and $\sigma_{r}^{2}$ indicates that the measurement occasion on average is a larger contributor to measurement variability than the measurement repetition. However, inspection of the interaction effect $\sigma_{slo}^{2}$ indicates that there is a considerable contribution of variability due to the measurement of a specific subject/leg on different occasions. We conclude that the palpation of landmarks and marking of the sensor’s position on the subject’s legs is one of the dominant sources of error and should therefore be taken into consideration for improvement of device design and measurement protocols. The highest contribution to the variance is the component $\sigma_{slor}^{2}$, indicating that for a specific subject/leg on a specific occasion, the different repetitions can show very high variability. This effect might be due to varying tibial geometries, soft-tissue artefacts or problems in aligning the sensor with the local anatomy. Importantly, the very similar results between the variance components between the inter-operator and intra-operator studies, together with the fact that the measurements by the different operators naturally also constitute different measurement occasions, indicate that changing the operator is not a key factor and contributes to only a small decrease in measurement reliability. We attribute the slight differences between the intra- and inter-operator studies to sampling variability and the smaller sample size of the inter-operator study, which increases the uncertainty of the respective estimates.

When comparing our results to the very similar study [16], in which 2 operators measured the right tibia of 10 subjects on 2 occasions, we find considerably higher variance components on all effects, even though the relative importance between them is consistent with the exception of the dominant noise contribution. Because the low amount of data collected by this study led to the variance components being estimated from as few as 2 values, this could have caused an underestimation of the true value of the variance components compared to our results. It is also possible, that the visual feedback available to the operator, in the form of the accelerations measured, made an efficient pre-selection of data for the two subjects possible and thereby reduced the variability.

The measurement procedure used to collect the data for the presented studies took approximately 10−15 min for both legs, and resulted in a generalizability coefficient of Φ = 0.67 (for determining the phase velocity for each leg. The data show that the reliability can be increased very efficiently by adding measurement occasions involving re-palpation and re-marking. For example, for a measurement procedure that measures one leg on two occasions, instead of two legs on one occasion, the generalizability coefficient rises to Φ = 0.80. Moreover, because of the low variance contributions of the effects involving m, the number of measurements per repetition can be reduced with minimal effect on the reliability, thus also reducing the required time for the measurement procedure. For example, reducing the number of measurements m from 50 to 1 only reduces the generalizability coefficient from Φ = 0.80 to Φ = 0.78.

Few other studies make allusions to the reliability of wave velocity measurements *in- vivo*. However, a study which measured 25 women on four days, reported the following ICCs: ICC(day-to-day) = 0.69, ICC(session-to-session) = 0.71, and ICC(trial-to-trial) = 0.89 [23], where the day corresponds to occasion, session corresponds to repetition, and trial corresponds to measurement for our study. Unfortunately, individual variance components were not reported, but the hierarchy of effects contributing to the variability agrees well with our findings, even though the frequency used in the experiment was slightly different. For another study, a coefficient of variation of 0.4% for ultrasound SOS measurements in human tibiae has been reported, which corresponds to ∼16 m/s [24]. One SOS measurement result in their study was created by taking 410000 velocity measurements as single measurement sequence. Only when three consecutive sequences were “statistically consistent”, which was not nearer specified, was the average of these three sequences considered a valid measurement result In another *in-vivo* study of ultrasound SOS measurements in human radii, intra-(inter-)operator precision of 15 m/s (20 m/s) has been reported, where the result of one measurement was obtained from measuring multiple waves, filtering outliers, and averaging three measurement sequences that were required to agree to within 1.5% of the measured value [25]. While missing details regarding measurement procedure, result selection, and result averaging procedure make an in-depth comparison with our study difficult, the reported results from these two studies agree well with our estimated standard error for a measurement procedure with a high number of repetitions r or occasions o (Fig 3). The high degree of agreement is likely a consequence of the measurement procedures in the reported studies employing a high number of measurements with some form of repositioning. This has a natural equivalent in our study of a measurement procedure employing a high number of occasions and repetitions. The agreement in absolute measurement error is interesting, as this occurs despite the different frequencies used.

In an *in-vitro* study on cadaveric radii, different methods of wave speed determination were compared and coefficients of variation of 0.5% and 0.4% were found for two SOS measurement procedures [9], results that agree well with the results from our study. For the measurement of the A0-similar mode at lower frequency (250 kHz and wave speeds (1300 m/s) a coefficient of variation of 2.7% was reported, which corresponds to ∼30 m/s. This number agrees well with the result ∼27 m/s from our study for a measurement procedure using single measurements (Fig 3). This fact is quite surprising, as *in-vitro* work without soft-tissue artefact is expected to be more reliable than *in-vivo* measurements using single measurement protocols. While one might think that the slightly different frequencies are responsible for the inflated coefficient of variation of the *in-vitro* measurement, comparison with studies using even higher frequencies indicates that the coefficient of variation actually decreases with increasing frequency. Here, further work is needed to assess the effects of different frequencies in a controlled and comparable manner. It is however quite plausible that our measurement procedure overestimates the reliability attainable for a single sensor, since it includes measurements from 4 sensors.

The grand-means of the measured phase velocities of agree very well with predictions from a Timoshenko and approximate Hermann-Mirsky theory for cylindrical shells [26], which have been shown to be valid models to describe many aspects of wave propagation in long bones [8, 12].The low slope of the linear regression of the velocity with respect to time indicates the absence of any relevant technical or operative drifts with time. This also supports the findings of other studies, suggesting that it is possible to use quantitative acoustics to assess longitudinal changes in study cohorts, e.g. training [17] or injury [27]. An interesting side product of this study are the results that demonstrate that wave velocities are significantly higher in males than in females. The fact that no statistically significant difference between the pooled velocities for the left versus the right leg of all subjects agrees with the results from studies, which showed no significant differences in the bone mass density between legs [28], with the exception of very homogenous elite-training groups [29].

## Conclusions

We have presented the first systematic quantification of the various sources of variability in the usage of axial-wave transmission, the results of which can be assumed to be generalizable to transmission based devices. The findings of this study indicate that care should be taken to ensure reproducible device positioning or to perform additional measurements with device repositioning in order to efficiently improve the reliability.
